# Comparative analysis of computed tomography severity indices in predicting the severity and clinical outcome in patients with acute pancreatitis

**DOI:** 10.12688/f1000research.125896.1

**Published:** 2022-11-08

**Authors:** Geetanjali Parmar, Griselda Philomena Noronha, Vinaya Poornima

**Affiliations:** 1 Department of Radiodiagnosis, Kasturba Medical College, Mangalore, Manipal Academy of Higher Education, Manipal, India., Mangalore, Karnataka, 575001, India

**Keywords:** Acute Pancreatitis, CT severity index, Modified CT severity index, clinical outcome parameters, Ranson’s criteria, hospital stay, multisystem organ dysfunction syndrome, sepsis.

## Abstract

**Background:** Acute pancreatitis (AP) has unpredictable severity. Its management is based on initial assessment of disease severity. It ranges from mild interstitial to severe necrotic form; the latter is associated with poor prognosis. Contrast-enhanced computed tomography (CT) of the abdomen is the gold standard in early detection of pancreatic necrosis and in assessing the severity of AP. Two CT grading systems exist to assess the severity of AP: CT severity Index (CSI) and modified CSI (MCSI). This study compares the usefulness of these two systems in predicting the severity and clinical outcome in AP in comparison with Ranson’s criteria and clinical outcome parameters.

**Methods:** This is a prospective hospital-based screening study of 80 patients aged >12 years with clinical diagnosis of AP who underwent contrast-enhanced CT study of the abdomen. Comparative analysis between MCSI and CSI with Ranson’s criteria and clinical outcome parameters was assessed by Chi-Squared test.

**Results:** The accuracy of CSI and MCSI in predicting the requirement of critical care, superadded infection, multiple organ dysfunction syndrome (MODS) and requirement of intervention were 73.0%, 64.5%, 69.8% 60.9% and 77.2%, 76.0%, 74.4% & 56.6% respectively. Area under the curve for MCSI score was significantly higher (AUC: 0.861; 95% CI: 0.736-0.986) than CSI score (AUC:0.815;95% CI:0.749-0.941). MCSI and CSI showed significant correlation with Ranson’s criteria; however, MCSI correlation was better (r:0.53; p<0.01) than CSI (r:0.35;p:0.04).

**Conclusion:** CSI and MCSI are better predictors of severity, clinical outcome and mortality compared with Ranson’s criteria, with MCSI being more accurate and better predictor than CSI. The accuracy of MCSI is better than CSI for prediction of requirement of critical care, development of superadded infection and development of MODS in AP. However, CSI and MCSI have low accuracy in predicting intervention in AP.

## Introduction

Acute pancreatitis (AP) is one of the most common causes of acute abdomen with unpredictable clinical course. Based on the severity, 80% of cases are mild and 20% of cases are severe which morphologically correlate with edematous and necrotizing forms of AP respectively. The mild form is self-limiting without causing major physiological insult. The severe form is life threatening and can lead to early or late multiple organ dysfunction syndrome (MODS) and superadded infection. ^1^^–^^3^

Contrast enhanced computed tomography (CT) of the abdomen is the gold standard ^4^ in identifying necrosis and fluid collections in AP which can aid in predicting disease severity and prognosis of the patient, thus guiding the management. Various studies suggest the evidence that severity can be better assessed by CT than the numerical grading systems due to direct visualization of necrosis and complications of AP on CT. ^5^^,^^6^ Although the CT severity index (CSI) shows good correlation with the severity of AP, few studies suggested few limitations. CSI doesn’t show good correlation with clinical outcome, mortality, need for surgical or percutaneous interventional procedures, MODS and superadded infection. ^3^ These shortcomings led to the modification and simplification of CSI by Mortele *et al*. ^3^ leading to the formation of modified CT severity index (MCSI). The present study is comparative analysis of MCSI and CSI with clinical outcome parameters and Ranson’s criteria in patients with AP.

## Methods

This is a prospective hospital-based screening study performed in the department of Radiodiagnosis affiliated to Kasturba Medical College (KMC), Mangalore (MLR), Manipal Academy of Higher Education (MAHE), Manipal, India on 80 patients with clinical diagnosis of AP who underwent contrast enhanced CT abdomen over a period of 2 years from September 2019 to September 2021. The study was performed after the approval from the Institutional Ethics Committee (IEC), KMC, MLR, MAHE with approval number of IEC KMC MLR 09-19/411.

Paediatric patients <12 years of age were excluded from the study, as Ranson’s criteria is not done in this group of patients in our hospital. Patients with poor imaging results due to poor compliance or motion artefacts were excluded. Patients without intravenous ( *i.v.*) contrast administration were also excluded. Patients with diagnosis of acute-on-chronic, recurrent and calcific pancreatitis and those that got discharged against medical advice or were lost to follow up were also excluded from the study. Patients with cardiac, renal & respiratory comorbidities were excluded from the study. Since the informed consent is routinely taken prior to every CT study and research data are obtained from the CT machine computer and patient case files with no direct interaction with the study participants, IEC, KMC, MLR waived off additional informed consent from the study participants for this research.

16-slice and 32-slice CT scanner machines were used to acquire 5-mm plain CT axial sections followed by the administration of 1.5–2.0 mL/kg body weight (80–100 mL) of non-ionic *i.v.* contrast through the automated injector. This was followed by around 1 mL/kg body weight (40–50 mL) of normal saline. The rate of injection for both contrast and saline administration was ~4 mL/s which was altered in accordance with haemodynamic status, body weight and size of the *i.v.* cannula. The images were acquired in the arterial and porto-venous phases at 6–8 and 35–45 seconds respectively in all cases by bolus tracking method which is described as follows. A locator was placed on the aorta at D12–L1 level and the contrast injection got automatically triggered *via* the automated injector once the aorta at this level showed optimum contrast opacification. Axial sections of 5 mm slice thickness were then reformatted to thin 0.6 mm axial, sagittal and coronal sections. The clinical and laboratory details of the patient were obtained from the CT requisition form and patient case file. This was followed by assessment of severity of acute pancreatitis using both CSI ( Tables 1a, 1b and 1c) and MCSI ( Tables 2a, 2b and 2c). Accordingly, severity of AP was graded as mild, moderate and severe based on the scores.

### CT severity index (CSI) ^7^

**Table 1a.** Grading of acute pancreatitis by CSI with allocation of points to each grade.

	CT findings	Points
**GRADE A**	Normal pancreas	0
**GRADE B**	Focal or diffuse enlargement of the pancreas including irregularity of gland contour, inhomogenous attenuation, dilatation of pancreatic duct and foci of small fluid collections within the gland, where there was no evidence of peri-pancreatic changes.	1
**GRADE C**	Abnormalities of pancreas which were intrinsic associated with hazy streaky densities representing inflammation in the surrounding peri-pancreatic fat.	2
**GRADE D**	A single ill-defined fluid collection (phlegmon).	3
**GRADE E**	Two or multiple, ill-defined collections of fluid or evidence of gas within or surrounding to the pancreas.	4

**Table 1b.** Assessment of presence & extent of pancreatic necrosis in AP by CSI with allotment of points.

Percentage of necrosis (%)	Points
Absent	0
<30	2
30–50	4
>50	6

**Table 1c.** Total points from CT grading of AP ( Table 1a) & assessment of pancreatic necrosis ( Table 1b) were combined to get CSI score with categorization of severity.

Severity of AP	CSI score
Mild	0 to 3
Moderate	4 to 6
Severe	7 to 10

### Modified CT Severity Index (MCSI) ^7^

**Table 2a.** Grading of acute pancreatitis by MCSI with allocation of points to each grade.

CT findings	Points
Normal pancreas	0
Intrinsic pancreatic abnormalities with or without inflammatory changes in peri-pancreatic fat	2
Pancreatic or peri-pancreatic fluid collection or peri-pancreatic fat necrosis	4

**Table 2b.** Assessment of presence & extent of pancreatic necrosis in AP by MCSI with addition of extra-pancreatic complications & allotment of points.

Percentage of necrosis (%)	Points
Absent	0
<30	2
>30	4
Extra-pancreatic complications (one or more of pleural effusion, ascites, vascular complications or gastrointestinal tract involvement)	2

**Table 2c.** Total points from CT grading of AP ( Table 2a) & assessment of pancreatic necrosis with extra-pancreatic complications ( Table 2b) were combined to get MCSI score with categorization of severity.

Severity of AP	MCSI score
Mild	0 to 2
Moderate	4 to 6
Severe	8 to 10

### Ranson’s criteria ^8^

Wherever available Ranson’s criteria score was noted down from patient case file and the correspondence of both the CT indices were studied with respect to the Ranson’s criteria. Ranson’s criteria score consists of 11 prognostic parameters, out of which five parameters are assessed at the admission and six parameters are assessed during initial 48 hours of hospital stay ( Tables 3a and 3b).

**Table 3a.** Assessment of five prognostic parameters of Ranson’s criteria at admission.

**Prognostic factors assessed at the time of admission**
Age more than 55 years
WBC Count more than 16,000 cells/mm ^3^
Blood Glucose more than 200 mg/dL
Serum glutamic oxaloacetic transaminase (AST) more than 250 U/L
Serum Lactate dehydrogenase (LDH) more than 350 U/L

**Table 3b.** Assessment of remaining six parameters of Ranson’s criteria during the first 48 hours of hospital stay.

Prognostic factors assessed during initial 48 hours of hospital stay
Serum calcium <8.0 mg/dL (<2.0 mmol/L)
Haematocrit fall > 10%
Arterial oxygen tension ( *P*O _2_) < 60 mmHg
Blood urea nitrogen increase by 5 mg/dL or more despite intravenous fluid hydration
Base deficit > 4 mEq/L
Sequestration of fluids >6 L

### Ranson’s score interpretation

Ranson’s score of 0 or 1 suggests complications will not develop in AP and mortality is negligible. On the other hand, Ranson’s score of 3 or more predicts severe AP with possible mortality. ^9^ The mortality in AP is directly proportional to the Ranson’s criteria score ( Table 3c).

**Table 3c.** Shows percentage of mortality with respect to the Ranson’s criteria score. ^10^

Ranson’s criteria score	Mortality (%)
0–2	0–3
3–4	15
5–6	40
7–11	100

The clinical outcome parameters ^6^^,^^7^ were noted down from all the patient case files and its association with CT severity indices were studied and are as follows:

1. The extent of hospital or intensive care unit (ICU) stay (greater than or equal to 15 days);
2. Requirement of critical care, (Arterial oxygen tension ( *P*O _2_) <60 mmHg or requirement of ventilation, systolic blood pressure (BP) <90 mmHg);
3. Requirement for (surgical/percutaneous) intervention (like drainage and aspiration);
4. Evidence of infection, (combination of a fever more than 100°F and elevated WBC count greater than 15,000 cells/mm);
5. Existence of organ failure (Arterial *P*O _2_ <60 mmHg or requirement of ventilation, serum creatinine of >3 mg/dL or urine output of <500 mL per 24 h and systolic BP of <90 mmHg); and
6. Death.

Outcome Variables that were studied are as follows:

• Sensitivity, specificity, positive predictive value, negative predictive value and accuracy of CSI and MCSI with respect to clinical outcome parameters like mean hospital stay, requirement of critical care, superadded infection, MODS, requirement of intervention & mortality.
• Concordance of CSI and MCSI with the score of Ranson’s criteria.

### Statistical analysis

The data was collected on a pre-designed study proforma. Qualitative data was expressed as percentage and frequency. Chi-Squared test was used to assess the association among the qualitative variables. The level of significance was represented by p-value of less than 0.05. Screening efficacy was computed using standard formulae. Wherever necessary, the results were graphically represented. Pearson correlation was used to assess the magnitude and direction of association between CSI and MCSI with Ranson’s score. Receiver operating characteristics (ROC) curves were used to compare the role of CSI and MCSI in predicting the mortality in AP with r value from +1 to −1. The r value of +0.1 to +1, 0 and −0.1 to −1 was suggestive of positive, zero and negative correlation respectively. Area under the curve (AUC) between CSI and MCSI as predictor of mortality was analyzed. Statistical package for social sciences (SPSS) version 21.0 (RRID:SCR_002865) and Microsoft Excel 2010 (RRID:SCR_016137) were used for most of the analysis and graphical representation respectively.

## Results

### Demographics

The patients with AP in this study were more or less equally distributed across all the decades from 2nd to 6th decade with mean age of 44.41 years. There was clear male predominance of 77.5% with 22.5% female patients with male to female ratio of 3.5:1. The most common cause for acute pancreatitis was alcoholism (56.3%) followed by gall stones (28.8%).

### Severity grading on CT by MCSI and CSI

As per MCSI, more than half of patients (56%) with AP had mild disease, about one third of them (36.3%) had moderate disease and a small percentage (7.5%) had severe disease ( Figure 1a).

**Figure 1a.**
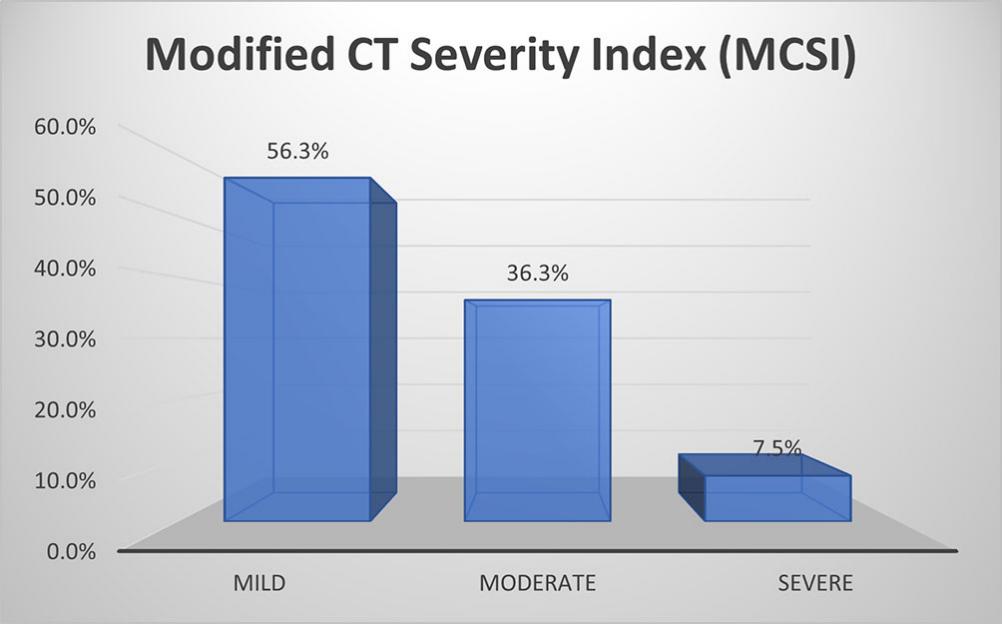
Bar diagram showing grading of AP by MCSI.

As per CSI, about half of patients (52.5%) with AP had moderate disease, about one fourth of them (26.3%) had mild disease and 21.3% had severe disease ( Figure 1b).

**Figure 1b.**
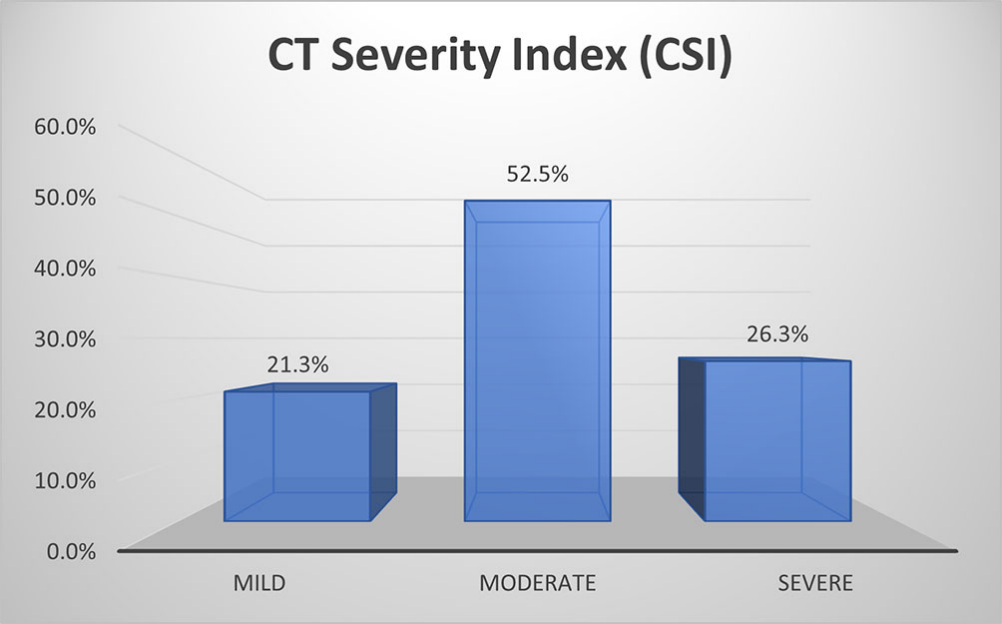
Bar diagram showing grading of AP by CSI.

### Association of MCSI and CSI score with clinical outcome parameters

**Requirement of critical care**

Based on MCSI score, all the patients with severe AP (100.0%) required critical care, 82.8% of moderate disease needed critical care and only one third of patients with mild disease (31.1%) needed intensive care with (p<0.01) ( Figure 2a). The overall sensitivity and specificity for prediction of requirement of critical care was 85.7% and 68.9% respectively with an accuracy of 77.2%.

**Figure 2a.**
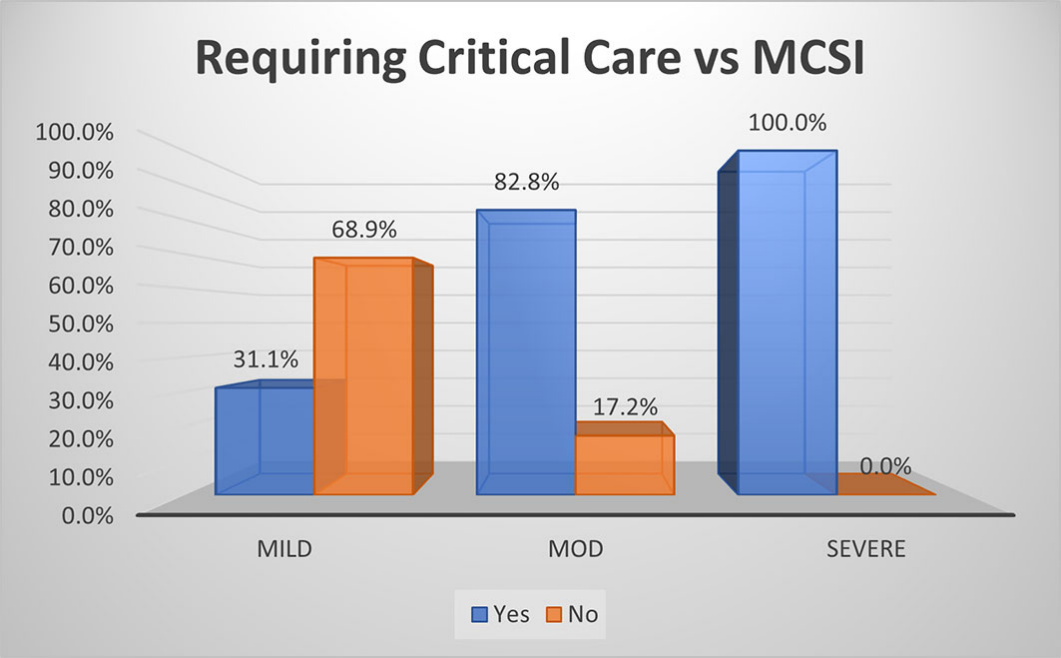
Bar diagram showing association of MCSI score with requirement of critical care.

As per CSI score, most (95.2%) of moderate disease, about half (52.4%) of severe disease and small percentage (11.8%) of mild disease required critical care (p<0.01) ( Figure 2b). The overall sensitivity and specificity for prediction of critical care requirement was 66.7% and 88.2% respectively with an accuracy of 73%.

**Figure 2b.**
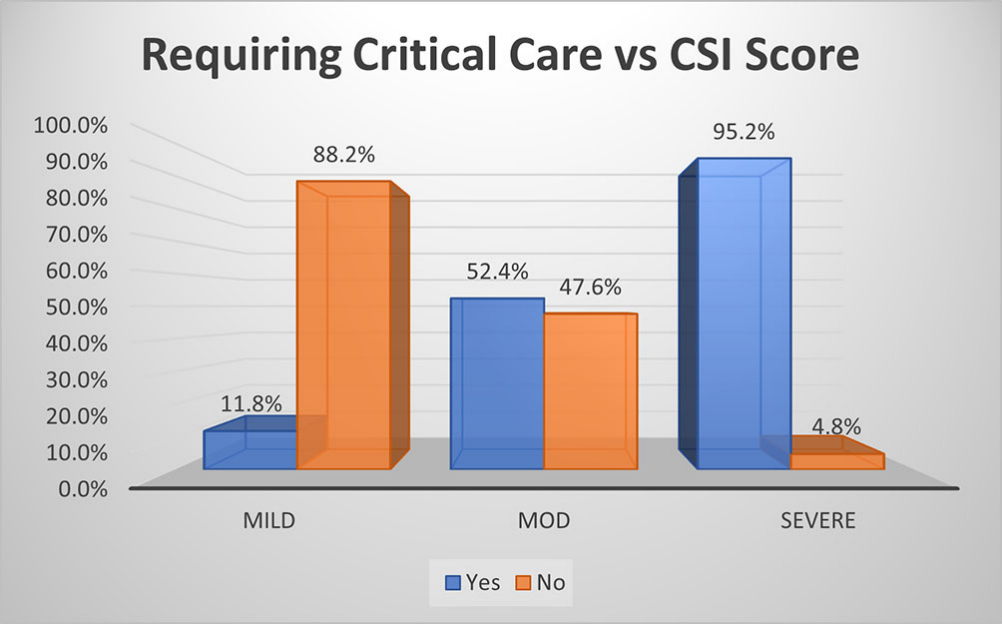
Bar diagram showing association of CSI score with requirement of critical care.

### Development of superadded infection

As per MCSI, the superadded infection was seen in 83%, 41% and 4% of severe, moderate and mild disease of AP respectively (p value<0.01) ( Figure 3a). The overall sensitivity and specificity were 49% & 96% respectively with an accuracy of 76% in predicting superadded infection in AP patients.

**Figure 3a.**
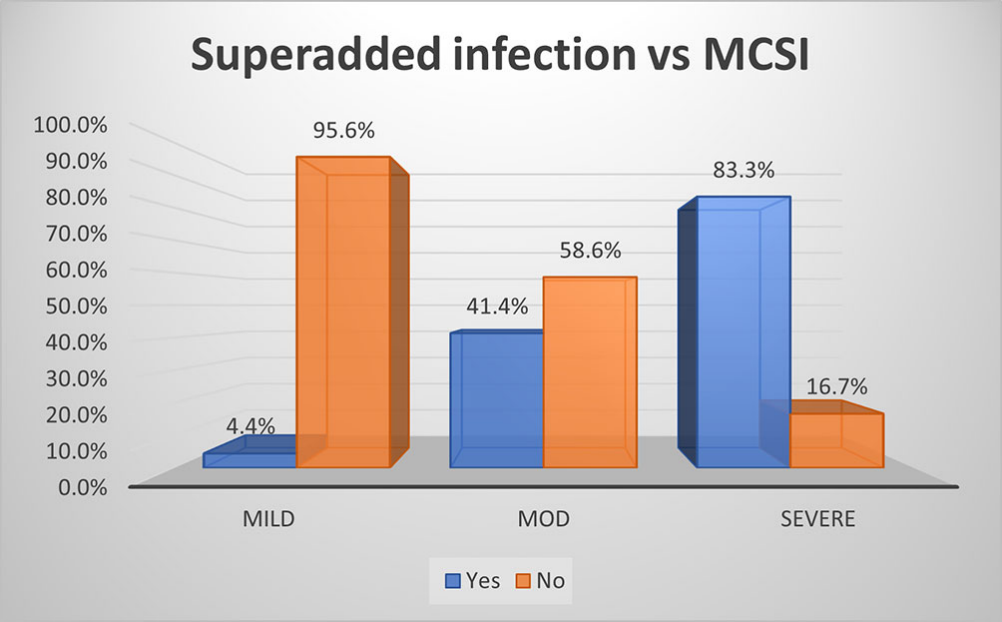
Bar diagram showing association of MCSI score with development of superadded infection.

Based on the CSI score, there was no (0.0%) superadded infection in mild disease, while it was present in slightly less than half (47.6%) of severe disease and about 21.4% in moderate disease (p<0.01) ( Figure 3b). Hence the overall specificity & sensitivity for prediction of presence of superadded infections was 100.0% and 30.2% respectively with accuracy of 64.5%.

**Figure 3b.**
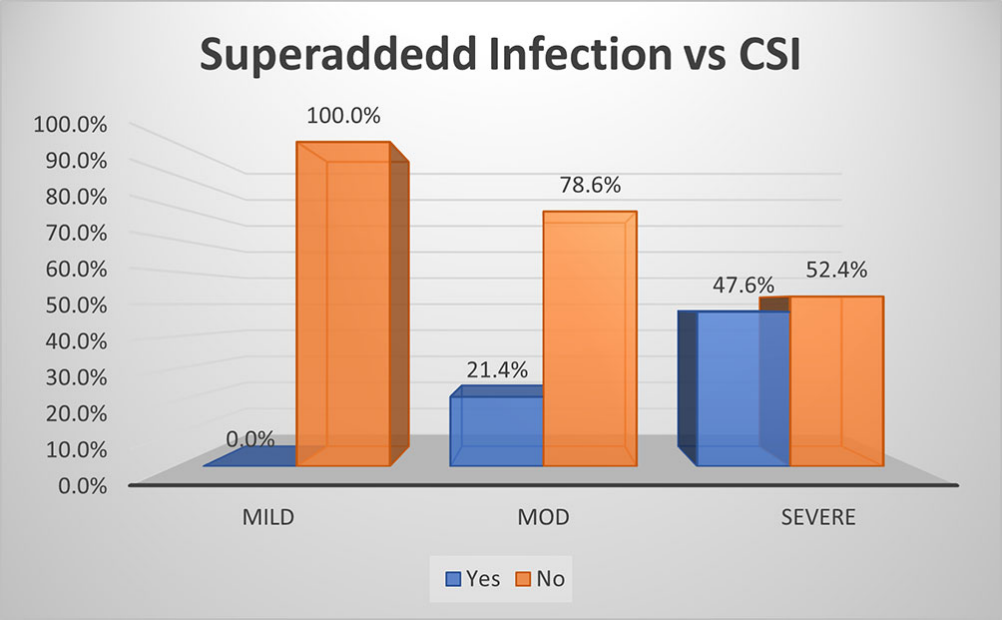
Bar diagram showing association of CSI score with development of superadded infection.

### Development of multiple organ dysfunction syndrome (MODS)

As per MCSI, MODS developed in 15.6% of mild, 58.6% of moderate and 83.3% of severe AP (p<0.01) ( Figure 4a). Overall sensitivity & specificity for prediction of development of MODS were 62.9% and 84.4% respectively with an accuracy of 74.4% respectively.

**Figure 4a.**
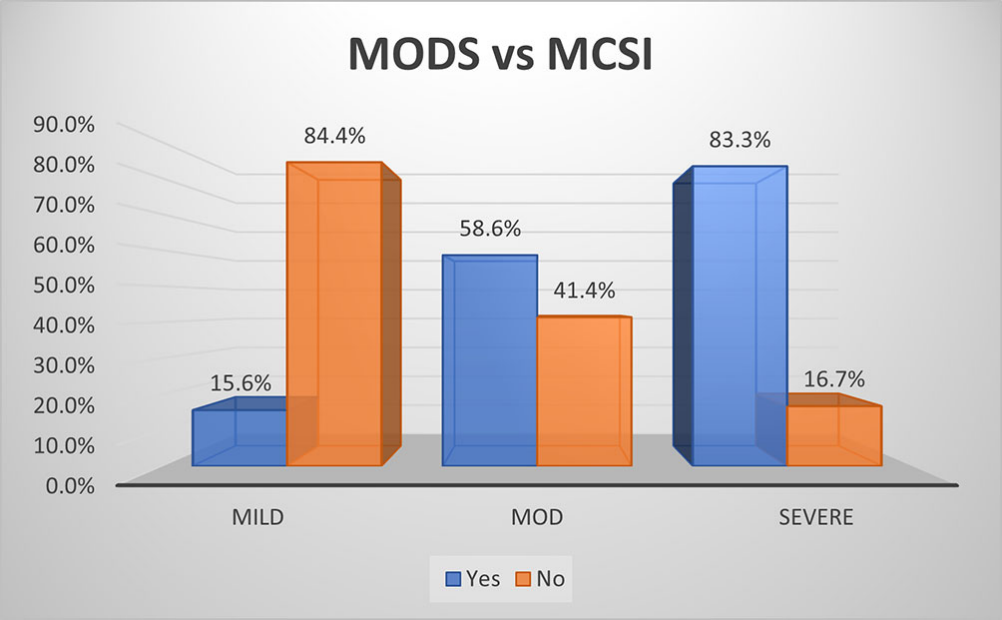
Bar diagram showing association of MCSI score with development of MODS.

As per the CSI score, there was no (0.0%) development of MODS in mild disease. On the contrary, most (95.2%) of severe disease and 21.4% percentage of moderate disease developed MODS ( Figure 4b). The overall specificity & sensitivity for prediction of development of MODS was 100.0% and 40.0% respectively with an accuracy of ~69.8%.

**Figure 4b.**
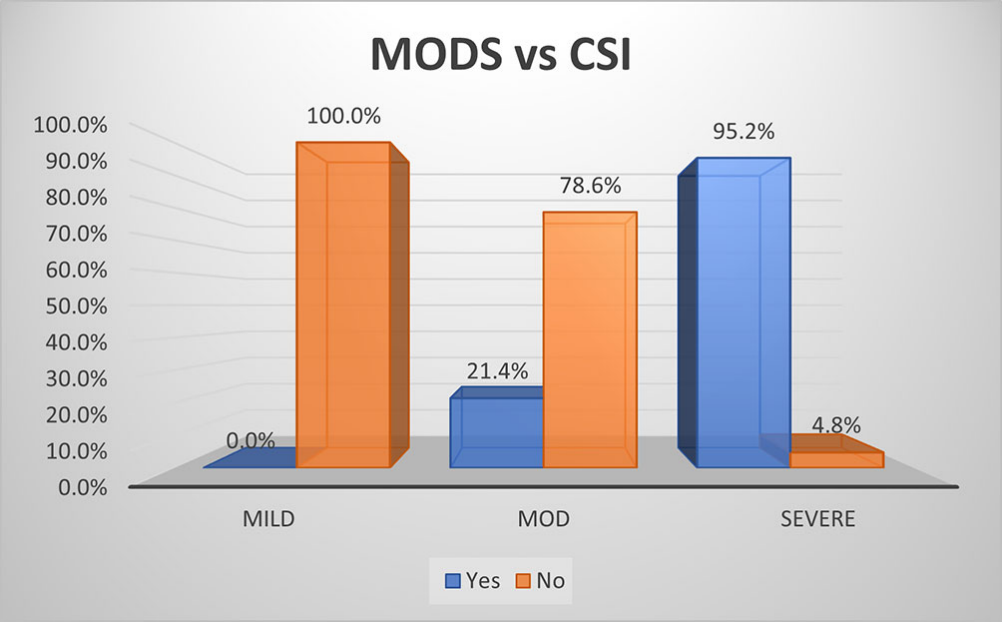
Bar diagram showing association of CSI score with development of MODS.

### Requirement of intervention

As per MCSI, intervention was performed in 55.6% of mild, 65.5% of moderate & 83.3% of severe cases of AP (p<0.01) ( Figure 5a). The overall sensitivity & specificity for prediction of requirement of intervention was 68.6% and 44.4% respectively with an accuracy of 56.6%.

**Figure 5a.**
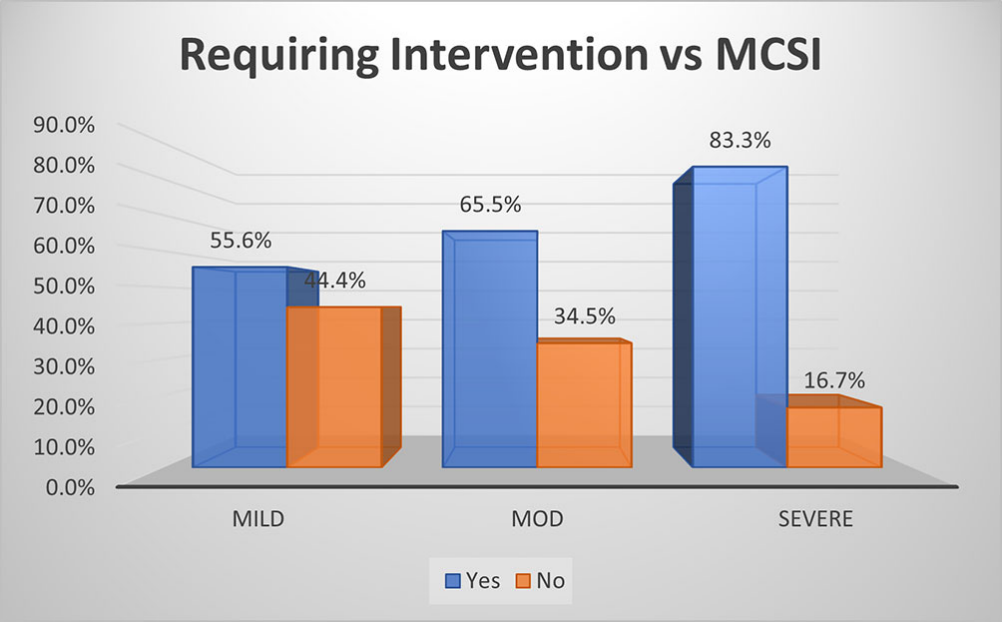
Bar diagram showing association of MCSI score with requirement of intervention.

As per CSI score, approximately three-quarters (76.2%) of patients with severe acute pancreatitis required intervention, 61.9% of patients with moderate disease and 41.2% of patients with mild disease required intervention (p<0.01) ( Figure 5b). The overall sensitivity and specificity for prediction of requirement of intervention by CSI was ~66.7% and ~58.8%, respectively, with an accuracy of ~60.9%.

**Figure 5b.**
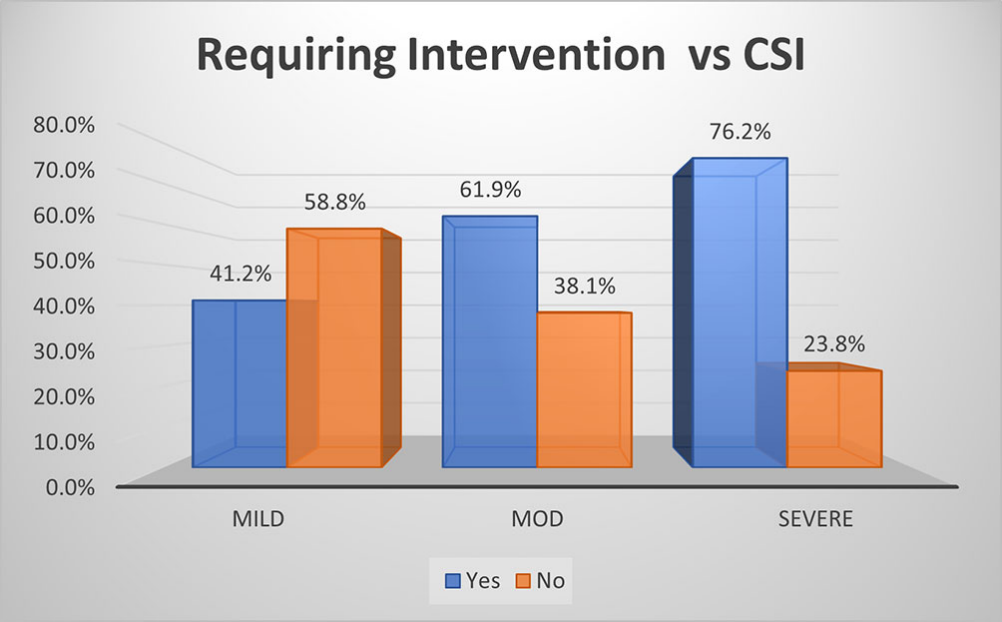
Bar diagram showing association of CSI score with requirement of intervention.

### Comparison of screening efficacy of MCSI and CSI with clinical outcome parameters

MCSI score showed both good sensitivity and specificity for development of MODS, good sensitivity for prediction of requirement of critical care & intervention. MCSI showed good specificity for the development of superadded infection ( Figure 6a).

**Figure 6a.**
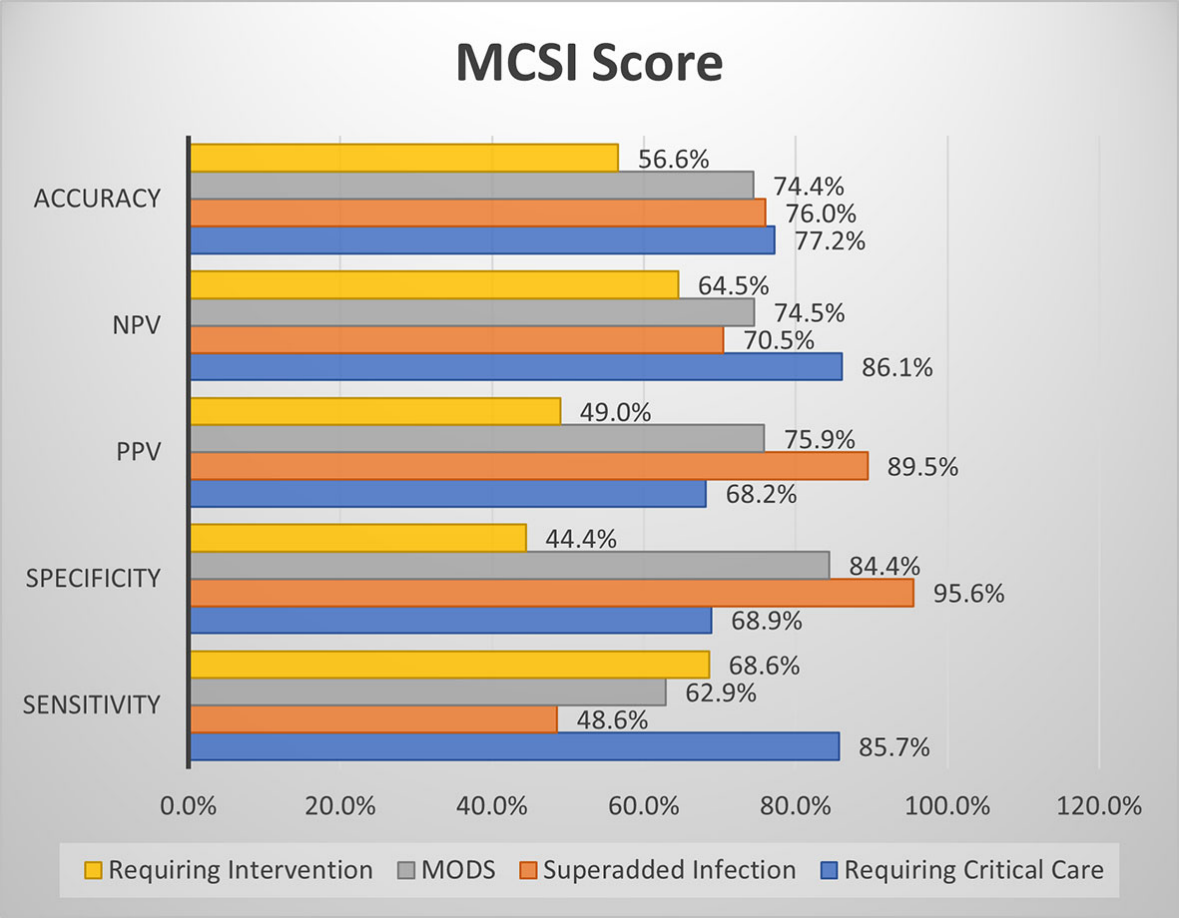
Shows screening efficacy of MCSI score with clinical outcome parameters.

CSI score showed high specificity for the development of MODS and superadded infection. The overall accuracy is better with MCSI score than CSI score for prediction of requirement of critical care, development of superadded infection & development of MODS. Both MCSI and CSI scores had low accuracy in predicting the requirement of intervention ( figure 6b).

**Figure 6b.**
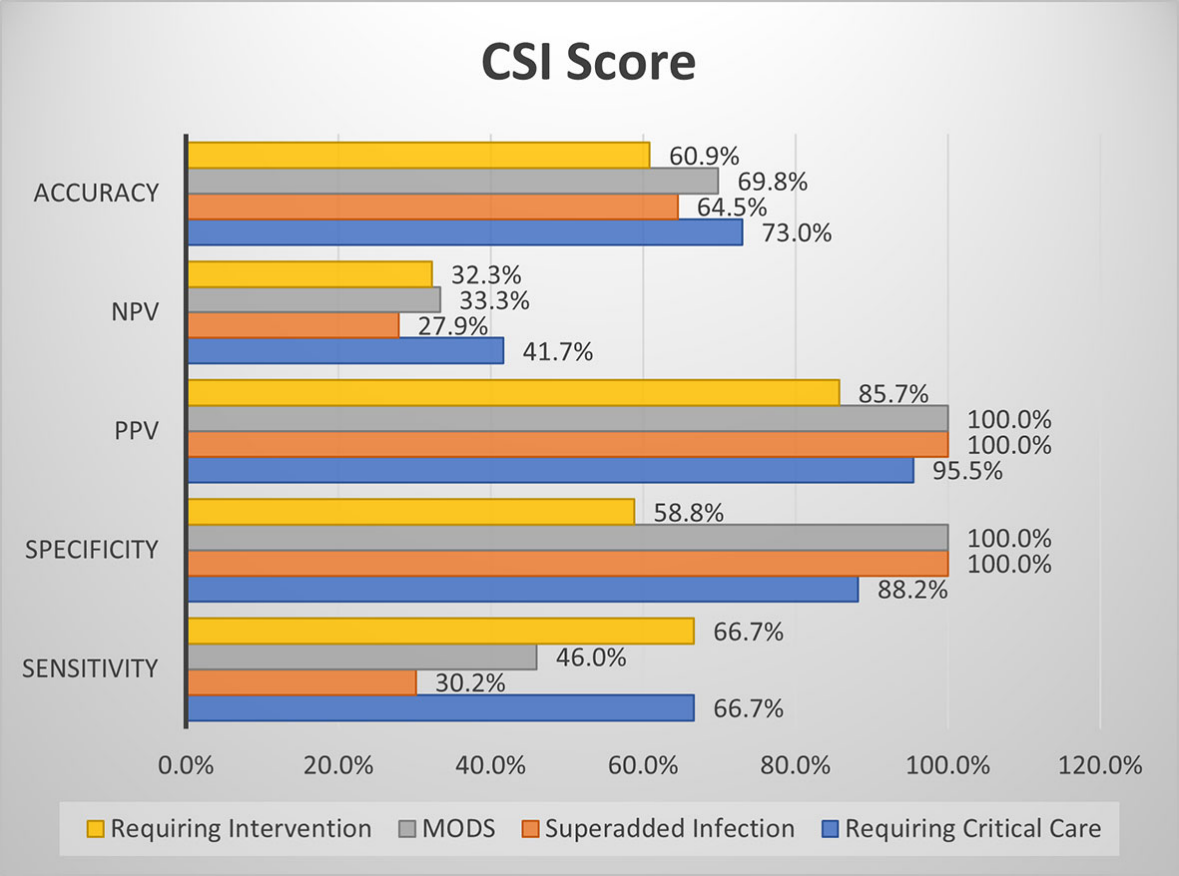
Shows screening efficacy of CSI score with clinical outcome parameters.

### Association of MCSI and CSI score with mortality

As per MCSI score, mortality rate was 100.0% in AP, 17.2% in moderate disease and 2.2% in mild disease ( Figure 7a). The overall sensitivity and specificity for prediction of mortality was 91.7% and 64.7%, respectively, with an accuracy of 87.5%.

**Figure 7a.**
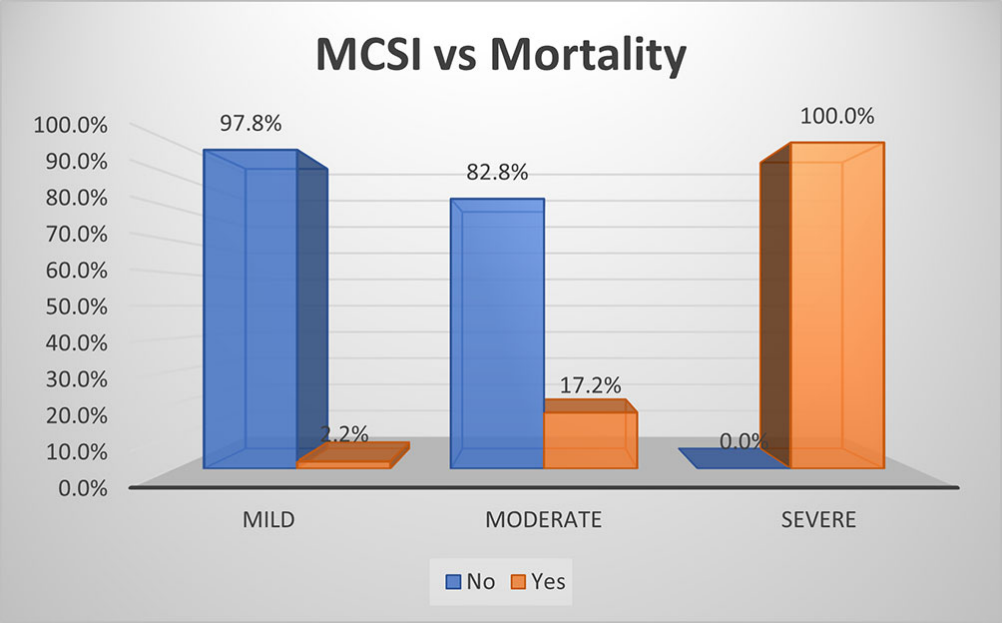
Bar diagram showing association of MCSI score with mortality.

As per CSI score, mortality rate was one-third (33.3%) and highest in severe grade of AP, followed by mild grade of AP (17.6%) with lowest mortality rate in moderate grade (4.8%) ( Figure 7b). The overall sensitivity & specificity for prediction of mortality was 58.5% and 79.4%, respectively, with an accuracy of 73.8%.

**Figure 7b.**
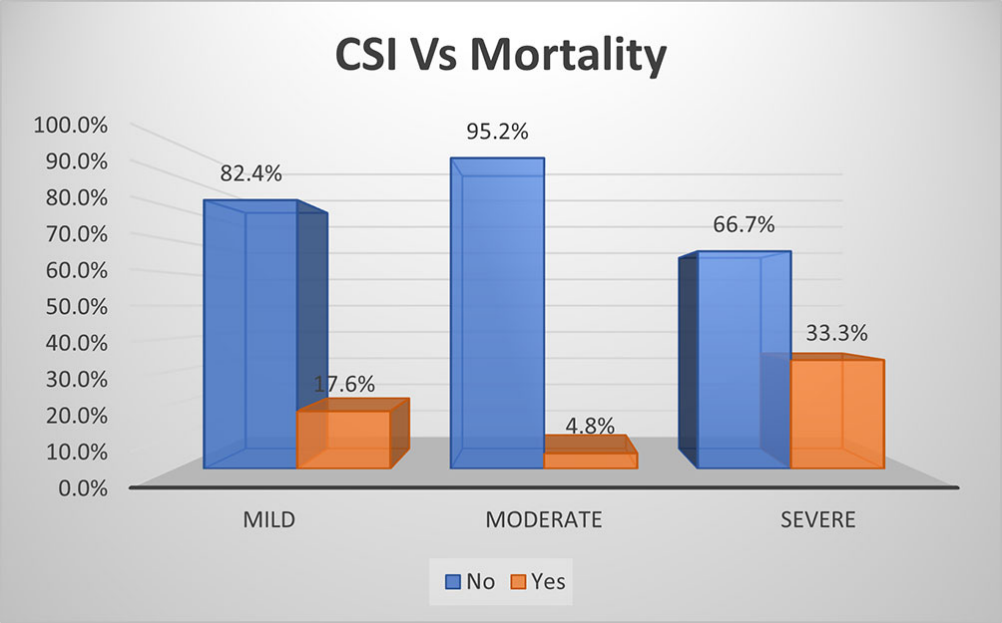
Bar diagram showing association of CSI score with mortality.

### Association of MCSI score with mean hospital stay

The mean hospital stay by MCSI was highest in moderate grade of AP with ~22 days as compared to approximately 12 & 13 days in mild & severe disease respectively (p<0.01) ( Table 4a).

**Table 4a.** Shows association of MCSI with mean hospital stay.

MCSI Score	N	Mean hospital stay (days)
**Mild**	45	12.27
**Moderate**	29	22.14
**Severe**	6	13.33
**Total**	80	15.93

The mean hospital stay as per CSI score was significantly higher in moderate and severe grade of acute pancreatitis corresponding to approximately 18 and 19 days respectively as opposed to approximately 8 days in mild disease (p<0.01) ( Table 4b).

**Table 4b.** Shows association of CSI with mean hospital stay.

CSI Score	N	Mean hospital stay (days)
**Mild**	17	7.53
**Moderate**	42	17.95
**Severe**	21	18.67
**Total**	80	15.93

### Correlation analysis for MCSI & CSI scores with Ranson’s criteria

Ranson’s criteria score was available with 31 out of 80 patients (38.8%). There was significant correlation between Ranson’s criteria and both CT severity indices (CSI and MCSI) but the correlation was highly statistically significant and better with MCSI score (r=0.53; p<0.01) as compared to CSI score (r=0.35; p=0.04) ( Table 5, Figures 8a & 8b).

**Table 5.** Shows Pearson correlation of MCSI & CSI scores with Ranson’s criteria score.

Pearson co-relation		
Ranson’s criteria	r-value	p-value
**CSI Score**	0.35	**0.040**
**MCSI Score**	0.53	**<0.01**

**Figure 8a.**
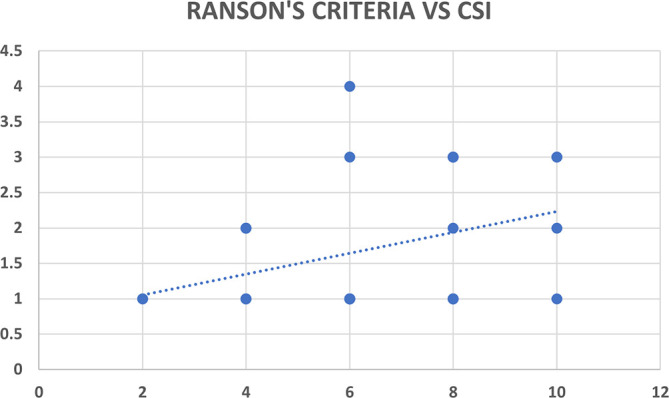
Scatter plot between CSI score (x axis) with Ranson’s criteria score (y axis) which shows positive correlation with Pearson correlation coefficient (r value) of 0.35.

**Figure 8b.**
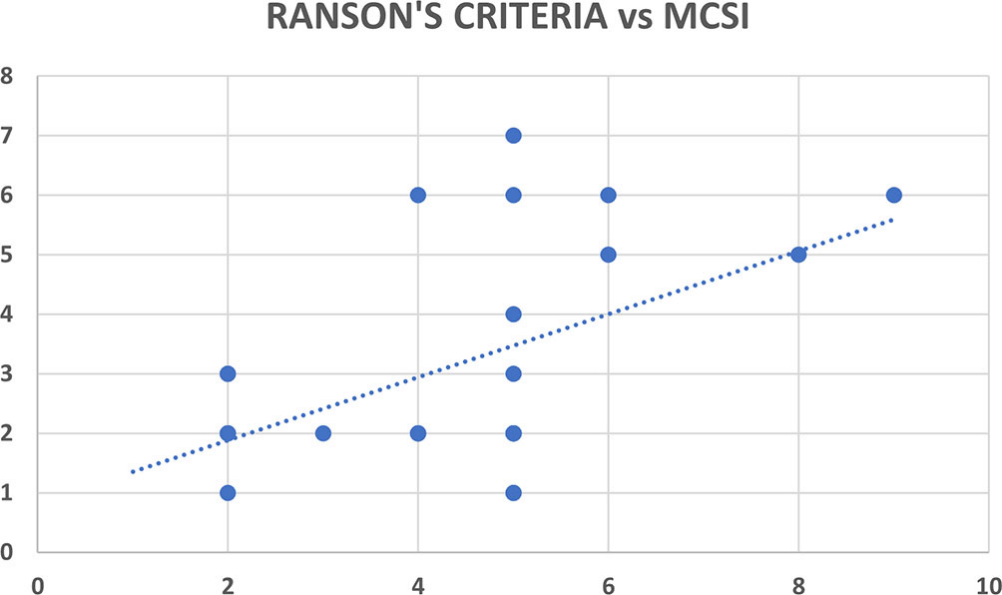
Scatter plot between MCSI score (x-axis) with Ranson’s criteria score (y-axis) which shows positive correlation with Pearson correlation coefficient (r value) of 0.53.

### ROC Curve analysis of MCSI & CSI score for prediction of mortality

**Figure 9.**
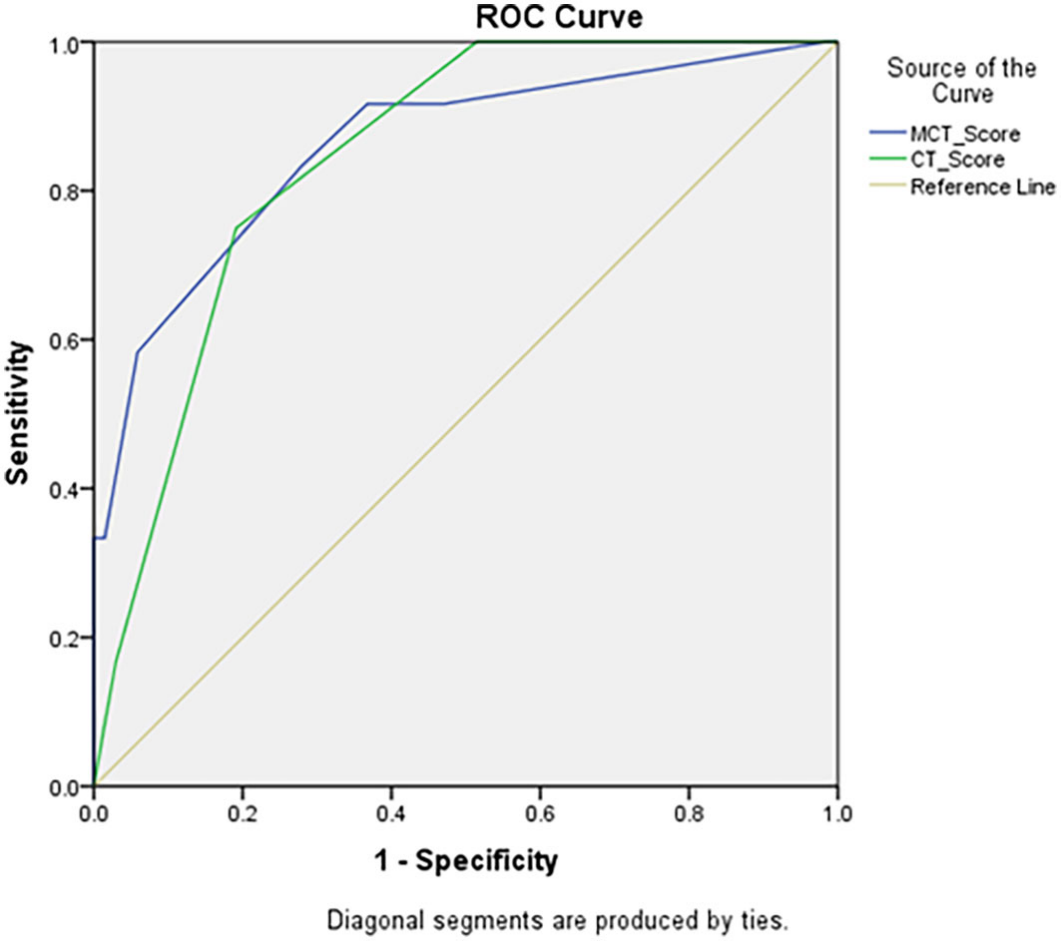
ROC curve analysis of CSI & MCSI scores shows both CSI (green coloured graph) & MCSI (purple coloured graph) as significant predictors of mortality in AP with MCSI score being better & more accurate than CSI score (yellow coloured graph is reference line).

According to ROC Curve analysis, both CSI & MCSI scores were significant predictors of development of mortality in AP. However, area under curve was significantly higher for MCSI score (AUC 0.861; 95% CI 0.736–0.986) as compared to CSI score (AUC 0.815; 95% CI 0.749–0.941) ( Table 6 & Figure 9).

**Table 6.** Shows area under the curve (AUC) analysis of CSI & MCSI in predicting mortality in AP.

Area under the curve					
Test result variable(s)	Area	SE	p-value	Asymptotic 95% confidence interval	
				Lower bound	Upper bound
**CSI Score**	0.815	0.049	<0.01	0.749	0.941
**MCSI Score**	0.861	0.064	<0.01	0.736	0.986

## Discussion

Contrast enhanced computed tomography of the abdomen is the imaging modality of choice and is the gold standard in the diagnosis of AP. The necrotizing form of AP, though less common, if present, is associated with a myriad of life-threatening complications. Among all the diagnostic tests available, CT has the highest diagnostic accuracy in detecting pancreatic necrosis. ^11^

Steinberg *et al.* ^12^ in their study suggested the evidence of 80 to 90% of AP was due to cholelithiasis & chronic alcoholism. Our study suggests evidence of alcoholism as the most common etiological factor for AP (56.3%) followed by gall stones (28.8%). Similar evidence was suggested by Wongnai *et al*. ^13^ in their study on 90 patients of AP, where alcoholism and pancreatico-biliary ductal calculi were reported as aetiological factor in 60% and 18% patients respectively. In India, alcohol consumption is predominantly seen in males (male to female ratio of 24.3:1). ^14^ The suggestive evidence of alcohol abuse as the commonest aetiological factor of AP combined with the male predominance of alcohol consumption in India explains the male to female preponderance (3.5:1) in this study. Similar evidence was suggested by Dugernier T L *et al*. ^15^ and Balthazar EJ *et al*. ^16^

On the contrary, Raghuwanshi S *et al*. ^17^ suggested the evidence of most common aetiology for AP as cholecystolithiasis (42%) followed by alcoholism (38%) with remaining 20% aetiology for AP belonged to rest category which included idiopathic, trauma and drug induced cases (24%, 2% and 2% respectively). Casas *et al*. ^18^ in their study on 148 patients suggested cholelithiasis (57%) as the most common aetiological factor for AP followed by alcoholism (21%) with both together contributing to another 5% of AP patients. Bollen TL *et al*. ^19^ and Jauregui *et al*. ^20^ also suggested the evidence of cholelithiasis as the predominant aetiological factor for AP.

### Severity of AP

This study is comparative analysis between MCSI and CSI grading systems in assessing severity and clinical outcome. Majority of the patients with AP belonged to mild category as per MCSI and moderate category as per CSI. This resulted in a small group of patients who had different category of severity by CSI and MCSI. The present study suggests MCSI to be more accurate predictor of severity than CSI as it predicted clinical outcome more accurately in those patients who were differently categorized in severity by CSI. This better prediction of severity and clinical outcome by MCSI in AP may be attributable to inclusion of extra-pancreatic complications of AP like ascites, pleural effusion, vascular complications and gastrointestinal complications in the assessment of MCSI which are not included in CSI. Kondekar S *et al*. ^7^ and Banday *et al*. ^21^ suggested partially opposing evidence from our study where majority of the patients by MCTSI belonged to mild category as per our study and the majority of the patients belonged to severe category as per CSI unlike our study.

### Clinical outcome parameters

Banday *et al*. ^21^ in their study suggested evidence of increasing mean duration of hospital stay with increasing severity by MCTSI score and concluded that the duration of mean hospital stay is directly proportional to severity grading by MCTSI system in acute pancreatitis.

Our study suggests mean hospital stay in AP by CSI score is significantly longer in moderate and severe disease as compared to mild disease (p<0.01) whereas the mean hospital stay by MCSI is significantly longer in moderate disease as compared to mild and severe disease (p<0.01). This can be attributed to the fact that mild cases were discharged relatively early from hospital in comparison to moderate category cases and very severe cases had higher mortality with lesser hospital stay.

Overall in the present study, MCSI score showed good sensitivity for prediction of requirement of critical care, development of MODS and requirement of intervention. MCSI showed good specificity for MODS and development of superadded infection. CSI showed high specificity for MODS and development of superadded infection. Overall accuracy of MCSI was better than CSI for prediction of requirement of critical care, development of superadded infection and development of MODS. Both scores showed lower accuracy with regard to requirement of intervention.

The sensitivity, specificity, positive predictive value (PPV) and negative predictive value (NPV) of MCSI in predicting severity according to the study by Bollen TL *et al*. ^19^ were 71%, 93%, 69% and 94%, respectively. This study suggested evidence of accurate correlation of clinical scoring systems with systemic complications & mortality in acute pancreatitis. The study also suggested evidence that radiological scoring system was more accurate in predicting the severity of acute pancreatitis, superadded infection and need for intervention than clinical scoring system. Among the two radiological scoring systems, the study suggested no evidence of significant differences between CSI and MCSI in predicting severity in acute pancreatitis.

Bollen *et al*. ^22^ suggested CSI showed better sensitivity, specificity, PPV and NPV than MCSI. Whereas Jauregui-Arrieta LK *et al*. ^19^ suggested different evidence where MCSI showed better sensitivity, specificity and PPV than CSI in severe AP and concluded that MCSI is better screening test than CSI in severe AP. Sharma *et al*. ^23^ performed suggested sensitivity and NPV is better with MCSI (98.6% and 90%, respectively) than CSI (87.3% and 57.1%, respectively) with similar PPV for both (~74%) and low specificity of 26.5% and 35.3% for MCSI and CSI, respectively.

### Ranson’s criteria

The present study shows significant correlation between Ranson’s criteria and both severity indices on CT (CSI and MCSI) but the correlation of MCSI with Ransons’ criteria is highly statistically significant which suggests MCSI as a better predictor of severity and clinical outcome than CSI.

On receiver operating characteristic (ROC) curve analysis, the present study suggests evidence that both CSI and MCSI are significant predictors of development of mortality in AP. However, the area under curve was significantly higher for MCSI score (AUC 0.861; 95% CI 0.736–0.986) as compared to CSI (AUC 0.815; 95% CI 0.749–0.941) which suggests MCSI as better predictor of mortality in AP than CSI.

Mangalanandan S *et al*. ^24^ suggested evidence of strong correlation between Ranson’s criteria and MCSI with mild and severe forms of AP showing 100% agreement with each other. But moderate category in MCSI Score had disagreeing results because Ranson’s criteria has only mild and severe categories due to which moderate category patients could not be studied. Their study suggested that MCSI (sensitivity of 93.33% and specificity of 54.17%) is more sensitive but less specific than Ranson’s criteria (sensitivity of 80% and specificity of 83.3%) in predicting actual outcome of AP. Although Chand P *et al*. ^25^ suggested evidence of lack of statistical significant difference between Ranson’s criteria and MCSI in evaluation of the outcome of AP with respect to the systemic complications, ^7^ there was statistically significant difference between MCSI and Ranson’s criteria with respect to local complications with increased incidence of local complications with higher Ranson’s criteria.

One of the drawbacks in the study was pediatric patients below the age of 12 years were not concluded as Ranson’s criteria is not done for them in our hospital. Also, larger sample size will reduce the margin of error in the study. Third drawback is the lack of availability of Ranson’s criteria score in 61.3% of the patients in the study due to usage of various other alternative clinical grading systems like Revised Atlanta Classification, Acute physiology and chronic health evaluation (APACHE) II & Bedside index of severity in acute pancreatitis (BISAP) by the treating clinician. These alternative clinical grading systems are affordable, quick & requires less effort in assessing the severity of AP than Ranson’s criteria.

## Conclusion

Both CSI and MCSI are better predictors of severity, clinical outcome and mortality than Ranson’s criteria in patients with AP with MCSI being more accurate & better predictor of the same than CSI. The accuracy of MCSI is better than CSI for prediction of requirement of critical care, development of superadded infection and development of MODS. Both CSI and MCSI scores have low accuracy with regard to requirement of intervention in AP patients.

## Data availability

### Underlying data

Mendeley: Underlying data for ‘Comparative analysis of computed tomography severity indices in predicting the severity and clinical outcome in patients with acute pancreatitis’, https://doi.org/10.17632/htkkzr9zbr.2. ^26^

Data are available under the terms of the Creative Commons Attribution 4.0 International license (CC-BY 4.0).
