# Supplementary material for: Comparative analysis of computed tomography severity indices in predicting the severity and clinical outcome in patients with acute pancreatitis
Source: F1000Res. 2024 Jul 18;11:1272. Originally published 2022 Nov 8. [Version 2] doi: 10.12688/f1000research.125896.2 (PMC11336560; doi:10.12688/f1000research.125896.2)
Supplement: Peer review history

# Reviewer report: Reviewer response for version 2

**DOI:** 10.5256/f1000research.168815.r304605

**Published:** 2024-08-20

**Authors:** Saurabh Dawra

**Affiliations:** 1 Postgraduate Institute of Medical Education and Research (PGIMER), Chandigarh, Punjab and Haryana, India

While the majority of queries have been addressed, I disagree that Atlanta criteria is based on CT findings. Infact it is a clinical criteria. 

 To my mind the major shortcomings of the study are: Single center study with limited sample size. Use of Ranson's criteria which is cumbersome, multiple investigations have to be done repeatedly and is no longer the standard of care at other center's. 

 Plz mention how useful is your study in present day scenario: Does it guide us to early/ late interventions, does it have prognostic utility to predict infections (ranson's criteria ) uses a lot of parameters). Does it guide towards early organ support ?

Is the work clearly and accurately presented and does it cite the current literature?

Partly

If applicable, is the statistical analysis and its interpretation appropriate?

Partly

Are all the source data underlying the results available to ensure full reproducibility?

No

Is the study design appropriate and is the work technically sound?

No

Are the conclusions drawn adequately supported by the results?

Partly

Are sufficient details of methods and analysis provided to allow replication by others?

No

Reviewer Expertise:

Pancreas, Liver

I confirm that I have read this submission and believe that I have an appropriate level of expertise to confirm that it is of an acceptable scientific standard, however I have significant reservations, as outlined above.

# Reviewer report: Reviewer response for version 2

**DOI:** 10.5256/f1000research.168815.r304604

**Published:** 2024-08-15

**Authors:** Juan Xiao

**Affiliations:** 1 Guilin Medical University, Guangxi, China

The authors have incorporated RANSON score in figure 9 (Table 6) which could be found that RANSON SCORE is better than MCSI Score or CSI Score in predicting mortality. RANSON score cutoff value got here could be used for analysis included RANSON score in figure 6.

Is the work clearly and accurately presented and does it cite the current literature?

Yes

If applicable, is the statistical analysis and its interpretation appropriate?

I cannot comment. A qualified statistician is required.

Are all the source data underlying the results available to ensure full reproducibility?

Yes

Is the study design appropriate and is the work technically sound?

Partly

Are the conclusions drawn adequately supported by the results?

Partly

Are sufficient details of methods and analysis provided to allow replication by others?

Yes

Reviewer Expertise:

NA

I confirm that I have read this submission and believe that I have an appropriate level of expertise to confirm that it is of an acceptable scientific standard, however I have significant reservations, as outlined above.

# Reviewer report: Reviewer response for version 1

**DOI:** 10.5256/f1000research.138249.r206452

**Published:** 2023-09-29

**Authors:** Juan Xiao

**Affiliations:** 1 Guilin Medical University, Guangxi, China

This study described the usefulness of modified CT severity index in the prediction of acute pancreatitis severity and outcome which is of clinical significance. However, there are still some issues which need to be addressed.

1. The authors should put the MSCI and SCI results compared to the same parameter together in one figure but not separately in Figure 1-5, 7.
2. The Ranson score result should be added in figure 6 and 9 in order to support your conclusion that MSCI is better than Ranscon score in outcome prediction.
3. Did the MSCI score correlate to the etiologies of acute pancreatitis? 

Is the work clearly and accurately presented and does it cite the current literature?

Yes

If applicable, is the statistical analysis and its interpretation appropriate?

I cannot comment. A qualified statistician is required.

Are all the source data underlying the results available to ensure full reproducibility?

Yes

Is the study design appropriate and is the work technically sound?

Partly

Are the conclusions drawn adequately supported by the results?

Partly

Are sufficient details of methods and analysis provided to allow replication by others?

Yes

Reviewer Expertise:

Biochemistry, baic medicine，acute pancreatitis

I confirm that I have read this submission and believe that I have an appropriate level of expertise to confirm that it is of an acceptable scientific standard, however I have significant reservations, as outlined above.

# Response

**Published:** 2024-03-15

**Authors:** Griselda Noronha

**Affiliations:** Kasturba Medical College Hospital, India

Dear Reviewer,

 Thank you for your detailed review of our article titled "Comparative Analysis of Computed Tomography Severity Indices in Predicting the Severity and Clinical Outcome in Patients with Acute Pancreatitis" submitted to the F1000 Journal. We appreciate your time and effort in providing constructive feedback to enhance the quality of our research.

 We acknowledge your suggestions.

 1) Based on your feedback, we will revise the figures accordingly to incorporate the MSCI and CSI results compared to the same parameter together in one figure but not separately in Figure 1-5, 7. We are committed to addressing all your concerns and making the necessary revisions to strengthen the validity and impact of our findings. Your valuable input will undoubtedly improve the clarity and reliability of our study, and we are grateful for your insightful suggestions.

 2) Regarding the addition of the Ranson score in Figures 6 and 9 of our manuscript to better support the conclusion that the MCSI is superior to the Ranson score in predicting clinical outcomes in patients with acute pancreatitis, we apologize for any confusion or oversight on our part in not including the Ranson score in the mentioned figures. It would be great if you could suggest how to incorporate the Ranson score in figure 6 and 9 as we are unable to figure it out. I discussed with my statistician and he was unable to figure it out. Hence your input would be greatly appreciated on this. 

 3) In our study we found that the most common etiology for acute pancreatitis was alcoholism followed by gall stones and third was idiopathic. we have not correlated MCSI with acute pancreatitis etiologies in our study.  

 We will promptly update the manuscript as per your recommendations and hope that these revisions will further enhance the significance and relevance of our research in the field of acute pancreatitis management.

 Thank you once again for your thorough review and for guiding us to refine our work for publication in F1000 Journal.

 Sincerely,

 Griselda Philomena Noronha

 Corresponding Author

# Reviewer report: Reviewer response for version 1

**DOI:** 10.5256/f1000research.138249.r206377

**Published:** 2023-09-29

**Authors:** Jan Trna

**Affiliations:** 1 Masaryk University, Brno, Czech Republic

Interesting topic with interesting results showing that CT results can predict severity well. This is probably not a completely new finding, however comparison with Ranson scoring system and showing that CTSI is better is interesting since CT is reproducible and not subjective. There are many new scoring systems and showing that good old CTSI is good is in my opinion a very good piece of evidence.

Is the work clearly and accurately presented and does it cite the current literature?

Yes

If applicable, is the statistical analysis and its interpretation appropriate?

Yes

Are all the source data underlying the results available to ensure full reproducibility?

Yes

Is the study design appropriate and is the work technically sound?

Yes

Are the conclusions drawn adequately supported by the results?

Yes

Are sufficient details of methods and analysis provided to allow replication by others?

Yes

Reviewer Expertise:

gastroenterology, endoscopy

I confirm that I have read this submission and believe that I have an appropriate level of expertise to confirm that it is of an acceptable scientific standard.

# Response

**Published:** 2024-03-15

**Authors:** Griselda Noronha

**Affiliations:** Kasturba Medical College Hospital, India

Thank you so much for your valuable comments and review report. Highly appreciate your feedback.

# Reviewer report: Reviewer response for version 1

**DOI:** 10.5256/f1000research.138249.r182028

**Published:** 2023-07-19

**Authors:** Saurabh Dawra

**Affiliations:** 1 Postgraduate Institute of Medical Education and Research (PGIMER), Chandigarh, Punjab and Haryana, India

Introduction:

1. I would recommend to change the first line that the disease has unpredicted severity. With the present level of research, we do have fair enough idea of predicted severity. The authors may rephrase the sentence.
2. While it is agreed that CTSI has it’s fair share of limitations, the same hasn’t been brought about clearly in the “Introduction“ section.
3. Please bring out clearly why have you chosen Hanson’s criteria as comparison to CTSI. What about Atlanta classification?

Methods:

1. How was pancreatitis diagnosed?
2. What is your institutional protocol of managing pancreatitis?
3. At what stage of disease/ day of illness was the CT done?
4. Do you perform all investigations as required to calculate Ranson’s criteria on all patients with AP?
5. What do you mean by critical care? What is your institute’s protocol in admitting the patients for critical care?

Discussion:

1. What is unique in your study? How is it different from other comparisons including meta analysis on the same topic?
2. What are your limitations?

Is the work clearly and accurately presented and does it cite the current literature?

Partly

If applicable, is the statistical analysis and its interpretation appropriate?

Partly

Are all the source data underlying the results available to ensure full reproducibility?

No

Is the study design appropriate and is the work technically sound?

No

Are the conclusions drawn adequately supported by the results?

Partly

Are sufficient details of methods and analysis provided to allow replication by others?

No

Reviewer Expertise:

Pancreas, Liver

I confirm that I have read this submission and believe that I have an appropriate level of expertise to state that I do not consider it to be of an acceptable scientific standard, for reasons outlined above.

# Response

**Published:** 2024-03-15

**Authors:** Griselda Noronha

**Affiliations:** Kasturba Medical College Hospital, India

Thank you for your insightful questions regarding our manuscript. We appreciate the opportunity to clarify these points and ensure a comprehensive understanding of our study protocols and findings. Below, please find detailed responses to each of your queries:

**introduction: **

1. **"I would recommend to change the first line that the disease has unpredicted severity. With the present level of research, we do have fair enough idea of predicted severity. The authors may rephrase the sentence"**- Thank you so much for bringing out this to us. we will surely rephrase this sentence. 
2. **"While it is agreed that CTSI has it’s fair share of limitations, the same hasn’t been brought about clearly in the “Introduction“ section"** - Again thank you for your valuable feedback. we shall do this change. 
3. **"Please bring out clearly why have you chosen Hanson’s criteria as comparison to CTSI. What about Atlanta classification?" **
  a)The Ranson criteria was the first scoring system specifically designed to assess the severity and prognosis of acute pancreatitis & hence it is extensively studied and validated globally. Since its used since ages, it is a reliable tool.
  b) Ranson’s score is directly correlated with patient outcomes which means it is a clear prognostic indicator. High score at admission and at 48hours later is associated with increased rate of mortality and morbidity which helps in stratifying patients according to the need for ICU admission and aggressive therapy.
  c) Ranson criteria is simpler and more straightforward, making it easier to apply in clinical settings, especially where immediate decisions are required while other scores like APACHE II requires more amount of data & is relatively complex to calculate.
  d) APACHE II and BISAP are non-specific to pancreatitis and are used in various critical care settings whereas Ranson’s criteria is specifically designed for acute pancreatitis.
  e) Though BISAP score is simple & easy to use, it predicts based on the assessment within first 24 hours of admission. It is relatively inferior in predicting the complications or mortality which can happen later in the course of illness. Ranson’s score is relatively superior to BISAP in this context as it considers assessment both at admission & 48hours after admission.
  f) APACHE II is more complex, need more data to calculate and it is not specific to acute pancreatitis as compared to Ransons score.  
4. **why not Atlanta Classification? **Atlanta classification is based on morphological assessment of acute pancreatitis severity on CT which usually takes places after atleast 48-72 hours. based on that we give CSI & MCSI scores.  Till then Ranson’s score will provide early prognostic information before detailed imaging findings are available.

**Method:**

**1. How was pancreatitis diagnosed?**

 The diagnosis of pancreatitis in our study followed a rigorous, multi-dimensional approach in line with established clinical guidelines. Initially, we considered the patient’s clinical presentation, including relevant history (e.g., alcohol consumption, gallstones), as well as symptoms like abdominal pain and vomiting. Objective findings such as ileus, fever, tachycardia, and hypotension were also critical. Crucially, we substantiated our clinical suspicion through laboratory evidence, particularly elevated lipase and amylase levels, which were at least threefold above the upper limit of normal. Finally, imaging played a pivotal role in our diagnostic algorithm. Contrast-Enhanced Computed Tomography (CECT) of the abdomen or Ultrasound was utilized to confirm the diagnosis, adhering to the principle that the clinician’s discretion, based on a synthesis of these data points, ultimately guided the diagnostic process.

  2. **Institutional protocol for managing pancreatitis**

 At our institution, the Department of Surgery manages pancreatitis cases. The management protocol begins at the point of presentation, where patients with symptoms indicative of pancreatitis or those evaluated for abdominal pain are assessed by the attending surgeon. Our approach prioritizes conservative management, including bowel rest, fluid resuscitation, and the administration of IV antibiotics and analgesics. Supportive measures such as supplemental oxygen, inotropic or ventilatory support, and transfusions for DIC are employed based on individual patient needs. Admission decisions—whether to the ward for mild cases or the ICU for those with significant organ dysfunction or unstable vitals—are made in accordance with the severity of the clinical presentation. Follow-up and further management, including imaging with CECT abdomen, are guided by established criteria, such as Ranson's criteria, and the clinical judgment concerning suspected complications.

 Reference :  (Banks PA, Freeman ML. Practice guidelines in acute pancreatitis. Am J Gastroenterol. 2006;101:2379–2400.)

  3. **At what stage of disease was CT done?**

 The timing for deploying CECT in our cases was carefully considered, primarily focusing on patients exhibiting signs of complicated pancreatitis, as defined by the Atlanta criteria, or when clinical suspicion of complications arose. Adhering to best practice, we generally deferred CT imaging for at least 48-72 hours following the onset of symptoms. This delay allows the acute inflammatory processes to stabilize, thereby enhancing diagnostic accuracy and informing subsequent management decisions, particularly in our ICU patients who are closely monitored for any signs of worsening or complications.

 Reference : Busireddy KK, AlObaidy M, Ramalho M, Kalubowila J, Baodong L, Santagostino I, Semelka RC. Pancreatitis-imaging approach. World J Gastrointest Pathophysiol. 2014 Aug 15;5(3):252-70. doi: 10.4291/wjgp.v5.i3.252. PMID: 25133027; PMCID: PMC4133524.)

 4) **Do you perform all investigations as required to calculate Ranson’s criteria on all patients with AP?**

      The investigations for Ranson's criteria were not performed for all cases but based on the clinical condition as per the discretion of the attending surgeon. Complete blood counts, glucose, calcium, liver and renal function tests and blood gas was performed at baseline for all cases whereas the post 48 hour investigations were done selectively based on the clinical condition of the patient. Based on the predicted severity, a decision to perform a CECT scan abdomen was taken.

 5. **What do you mean by critical care? What is your institute’s protocol in admitting the patients for critical care?**

 We had a Surgical intensive care unit at our hospital with ventilators and routine intensive care support systems. Unfortunately, there was no step-up or high dependency unit (HDU) facility available. Any patient who was found to have any organ dysfunction or features of shock was admitted to the ICU for monitoring and treatment and started on appropriate treatment. They were provided with supplemental oxygen, Non invasive ventilation (NIV) or endotracheal intubation with ventilation as and when required and hemodynamic support with transfusions or inotropic and pressor supports along with routine conservative management.

**Discussion:**

 6) **unique in our study :**

 There was no other study in the literature comparing both radiological severity indicators CSI & MCSI with Ranson's score alone in this combination at the time of conceptualization of this study. there were studies comparing CSI alone or MCSI alone with Ranson score or correlating radiological scores (CSI/MCSI) with multiple clinical scoring systems. 

 7) **Limitations of study :**

 a) the patients who underwent CECT for acute pancreatitis but who were discharged against medial advice or lost for follow up could not be assessed and excluded from the study.

 b)the study did not include patients below 12 years of age as Ranson's criteria is not done for paediatric patients in our hospital. 

 c)smaller sample size which may increase the margin of error when compared to a study with a relatively larger sample size. 

 d) Since no single scoring system can universally predict the patient outcome perfectly, it is a limitation of the study. 

 e) Ranson's score needs 48hours to complete the score. CSI & MCSI is obtained after atleast 48-72hours of admission. So there is delay of obtaining scores and hence it has lesser sensitivity in very early stages of disease. 

 We trust these responses address your queries comprehensively. Our team is committed to delivering research that meets the highest standards of clinical rigor and transparency. Should you require further details or clarification, we are at your disposal.
